# HAT regimen attenuates NF-κb-driven megakaryocyte apoptosis and neuronal cell death in sepsis: convergent mechanisms protecting thrombocytopenia and cognitive function

**DOI:** 10.3389/fcell.2026.1880883

**Published:** 2026-07-20

**Authors:** Zhi Hui, Na Shen, Hewei Zhang, Min Yu, Zhe Li, Jialu Ping, Qiang Fu

**Affiliations:** 1 The Fourth Central Clinical School, Tianjin Medical University, Tianjin, China; 2 Department of Critical Care Medicine, Cangzhou Central Hospital, Cangzhou, China; 3 Central Laboratory, Tianjin Fourth Central Hospital, Tianjin, China; 4 Department of Critical Care Medicine, Tianjin Fourth Central Hospital, Tianjin, China; 5 Department of Emergency Medicine, Rizhao People’s Hospital, Rizhao, China; 6 Department of Cardiovascular Prevention and Rehabilitation, Cangzhou Central Hospital, Cangzhou, China; 7 Department of Critical Care Medicine, Tianjin Haihe Hospital, Tianjin, China

**Keywords:** apoptosis, cell survival, HAT regimen, megakaryocyte, neuroinflammation, neuronal cell death, NF-κB signaling, programmed cell death

## Abstract

**Background:**

Sepsis-induced NF-κB hyperactivation drives two devastating cell death cascades: megakaryocyte apoptosis causing thrombocytopenia (incidence 35%–59%), and neuronal apoptosis with microglial-mediated neuroinflammation causing cognitive dysfunction in up to 70% of survivors. Mechanistically, NF-κB–driven upregulation of pro-apoptotic mediators (cleaved caspase-3, cytochrome c release) impairs megakaryopoiesis while simultaneously inducing hippocampal neuronal death and synaptic loss. The HAT regimen (hydrocortisone, ascorbic acid, and thiamine) modulates complementary nodes of this NF-κB/apoptosis axis, yet its cell-autonomous mechanisms of protecting megakaryocytes and neurons from sepsis-induced programmed cell death remain uncharacterized.

**Methods:**

We employed a multi-level translational approach to interrogate HAT-mediated cell survival mechanisms. A retrospective cohort of 184 propensity score-matched sepsis patients with thrombocytopenia provided clinical validation. Mechanistic studies used cecal ligation and puncture (CLP) in C57BL/6 mice, with cell death profiling (TUNEL, cleaved caspase-3, Annexin V), blood-brain barrier integrity assays, and synaptic protein quantification. *In vitro* apoptosis and proliferation assays used MEG-01 megakaryoblasts as a preliminary screening platform (note: MEG-01 harbors BCR-ABL, which constitutively elevates baseline NF-κB activity; primary mechanistic conclusions are grounded in the CD34^+^ primary system) and primary CD34^+^ hematopoietic stem cells, BV-2 microglia, and primary hippocampal neurons, combined with NF-κB pathway dissection (p65 nuclear translocation, IKK phosphorylation, IκBα dynamics), genetic validation by p65 siRNA knockdown, and transcriptomic/proteomic profiling, delineated component-specific pro-survival mechanisms. Pharmacological synergy was formally quantified by Chou-Talalay CI analysis. E-value sensitivity analyses were applied to primary clinical endpoints.

**Results:**

HAT synergistically suppressed NF-κB p65 nuclear translocation by 52% in megakaryoblasts and 54% in hippocampal tissue, reducing pro-inflammatory cytokines by 35%–42%. In megakaryocytes, HAT inhibited apoptosis by 48.6% and enhanced proplatelet formation by 52.4%, corresponding to accelerated platelet recovery (78.5% vs. 42.3% increase at day 7, P < 0.001) and reduced 28-day mortality (22.8% vs. 34.8%, P = 0.038) in patients. In the CLP model, HAT reduced hippocampal neuronal apoptosis (TUNEL+ cells −54.8%; cleaved caspase-3+ neurons −58.2%), attenuated microglial activation (Iba-1+ cells −48.6%), preserved blood-brain barrier integrity (Evans blue extravasation −62.4%), and maintained synaptic protein expression (PSD-95 + 42.6%; synaptophysin +38.4%), translating to significant cognitive and psychological benefit in survivors. Each HAT component contributed distinct anti-apoptotic mechanisms—hydrocortisone suppressed p65 translocation, ascorbic acid blocked ROS-mediated IKK activation, and thiamine restored mitochondrial membrane potential—producing formally synergistic (Chou-Talalay CI = 0.61 in megakaryocytes and CI = 0.58 in hippocampal neurons, both <1.0) pro-survival effects exceeding individual component efficacy. HAT group membership remained an independent predictor of preserved cognition on multivariable regression adjusting for ventilation duration and ICU stay (adjusted OR 0.38, 95% CI 0.18–0.79, P = 0.009).

**Conclusion:**

HAT therapy protects two distinct cell populations—megakaryocytes and hippocampal neurons—from sepsis-induced programmed cell death through convergent, component-specific suppression of NF-κB–driven apoptotic signaling. These mechanistic findings reframe HAT as a broad-spectrum anti-apoptotic intervention, providing a cellular and molecular rationale consistent with its dual clinical benefit against thrombocytopenia and cognitive dysfunction in sepsis. Given the null findings of major HAT RCTs in unselected populations, these mechanisms particularly support biomarker-enriched trial designs.

## 1 Introduction

Sepsis, defined as life-threatening organ dysfunction caused by a dysregulated host response to infection, is fundamentally a disease of pathological cell death. The systemic inflammatory cascade triggers programmed death across multiple cell lineages through NF-κB–dependent upregulation of pro-apoptotic mediators, resulting in organ dysfunction and failure (Singer et al., 2016). Approximately 48.9 million sepsis cases occur annually worldwide, resulting in 11 million deaths (Rudd et al., 2020). While the immunological dysregulation of sepsis has been extensively characterized, the cell type–specific mechanisms driving NF-κB–mediated apoptosis in distinct cell populations, and therapeutic strategies capable of simultaneously protecting multiple cell lineages, remain insufficiently understood.

Two prominent cell death phenotypes define the most clinically consequential complications of sepsis. First, megakaryocyte apoptosis and impaired megakaryopoiesis underlie sepsis-associated thrombocytopenia, which affects 35%–59% of patients (Venkata et al., 2013). NF-κB activation in bone marrow megakaryocytes upregulates pro-apoptotic Bcl-2 family members and caspase cascades while suppressing thrombopoietin receptor signaling, collectively blocking proplatelet formation and platelet biogenesis (Lee et al., 1993; Claushuis et al., 2016). The resulting thrombocytopenia independently predicts mortality, bleeding, and prolonged ICU stay (Lee et al., 1993). Central to these pathological processes is aberrant NF-κB signaling, which orchestrates the inflammatory cascade and directly impairs megakaryopoiesis (Mussbacher et al., 2019).

Second, sepsis-induced neuronal cell death and glial activation drive sepsis-associated encephalopathy (SAE), with up to 70% of survivors exhibiting persistent cognitive dysfunction (Gofton and Young, 2012). At the cellular level, NF-κB hyperactivation in microglia promotes a pro-inflammatory M1 phenotype, with secretion of TNF-α, IL-1β, and reactive oxygen species that execute bystander hippocampal neuronal apoptosis via the intrinsic mitochondrial pathway (Sonneville et al., 2021; Liu et al., 2017). Concurrently, blood-brain barrier disruption facilitates peripheral cytokine entry, amplifying the neuronal death cascade. Dendritic spine loss and synaptic protein degradation—downstream consequences of caspase-3 activation in neurons—underlie the memory and executive function deficits that persist long after ICU discharge (Iwashyna et al., 2010). The NF-κB pathway plays a pivotal role in mediating these neuroinflammatory responses, promoting microglial polarization toward pro-inflammatory phenotypes, and inducing neuronal apoptosis (Liu et al., 2017).

The convergence of NF-κB–driven apoptosis in both megakaryocytes and neurons as core mechanisms underlying two major sepsis complications raises a compelling question: can a single therapeutic strategy simultaneously protect these divergent cell populations through their shared pro-death signaling node? The HAT regimen, comprising hydrocortisone, ascorbic acid (vitamin C), and thiamine, represents a mechanistically rational candidate. Each component engages distinct upstream regulators of NF-κB activation and mitochondrial apoptotic priming: hydrocortisone via glucocorticoid receptor-NF-κB cross-talk, ascorbic acid via antioxidant suppression of IKK kinase complex, and thiamine via restoration of mitochondrial metabolic homeostasis (Marik et al., 2017). This combination was first proposed by Marik and colleagues, who reported mortality reduction in severe sepsis and septic shock (Marik et al., 2017). However, whether HAT exerts direct anti-apoptotic, pro-survival effects at the cellular level in megakaryocytes and neurons, and the molecular hierarchy governing these effects, has never been systematically examined.

Hydrocortisone, a glucocorticoid with potent anti-inflammatory effects, acts by inhibiting NF-κB nuclear translocation through induction of IκBα synthesis and suppressing the transcription of pro-inflammatory genes (Auphan et al., 1995). In sepsis, hydrocortisone helps restore vascular tone, reduce vasopressor requirements, and attenuate the cytokine storm (Annane et al., 2017). Ascorbic acid functions as a powerful antioxidant that scavenges reactive oxygen species, thereby preventing oxidative activation of the IκB kinase complex and subsequent NF-κB activation (Cárcamo et al., 2004). Additionally, vitamin C supports endothelial barrier function, enhances immune cell function, and serves as an essential cofactor for catecholamine synthesis. Thiamine, a water-soluble B vitamin, is critical for mitochondrial oxidative metabolism and ATP production through its role as a cofactor for pyruvate dehydrogenase in the Krebs cycle (Donnino et al., 2010). Thiamine deficiency, which is prevalent in critically ill patients with sepsis, impairs cellular energy metabolism and exacerbates oxidative stress, thereby potentiating NF-κB activation and inflammatory responses.

Despite the biological plausibility and promising preliminary clinical data supporting HAT therapy, its specific effects on sepsis-associated thrombocytopenia and cognitive dysfunction have not been systematically investigated. Furthermore, the molecular mechanisms by which these three agents may synergistically modulate the NF-κB pathway to protect megakaryocyte function and prevent neuroinflammation remain poorly understood. Elucidating these mechanisms is essential for optimizing therapeutic strategies and identifying patients most likely to benefit from this intervention.

The present study was designed to address these knowledge gaps through a comprehensive translational research approach. We conducted a retrospective clinical study to evaluate the effects of HAT therapy on platelet recovery and clinical outcomes in sepsis patients with thrombocytopenia. Concurrently, we employed a murine cecal ligation and puncture model to assess the impact of HAT treatment on cognitive function, neuroinflammation, and synaptic integrity. Additionally, *in vitro* experiments using human megakaryocyte cell lines and primary hematopoietic stem cells were performed to delineate the molecular mechanisms underlying HAT-mediated protection of megakaryopoiesis. Through this integrated approach, we aimed to provide robust evidence supporting the clinical application of HAT therapy and to establish a mechanistic framework for understanding its therapeutic benefits in sepsis-associated thrombocytopenia and cognitive dysfunction.

## 2 Methods

### 2.1 Clinical study design

This retrospective cohort study reviewed medical records from intensive care units across multiple tertiary hospitals between January 2020 and December 2024. The study was approved by the Institutional Review Board of each participating center with waiver of informed consent.

Inclusion criteria comprised adult patients (aged 18–80 years) meeting Sepsis-3 diagnostic criteria with concurrent thrombocytopenia (platelet count <150 × 10^9^/L). Exclusion criteria included pre-existing hematological malignancies, chronic liver cirrhosis, active malignancy receiving chemotherapy, pregnancy, pre-existing neurodegenerative diseases, and psychiatric disorders.

Patients were classified into the HAT treatment group (receiving hydrocortisone 50 mg every 6 h, ascorbic acid 1.5 g every 6 h for 4 days, and thiamine 200 mg every 12 h for at least 4 days) or the control group (standard sepsis care alone). Propensity score matching was performed using baseline characteristics including age, sex, comorbidities, SOFA score, and baseline platelet count to minimize selection bias.

The primary outcome was percentage change in platelet count from baseline at days 3 and 7. Secondary outcomes included time to platelet recovery, transfusion requirements, bleeding events, inflammatory biomarkers, and clinical outcomes (28-day mortality, ICU length of stay, duration of mechanical ventilation).

### 2.2 Cognitive and psychological assessment

Surviving patients attending follow-up visits at 3 and 6 months post-discharge underwent comprehensive neuropsychological evaluation. Global cognitive function was assessed using the Montreal Cognitive Assessment (MoCA) and Mini-Mental State Examination. Domain-specific assessments included the Trail Making Test for attention and executive function, Rey Auditory Verbal Learning Test for memory, and Digit Symbol Substitution Test for processing speed.

Psychological outcomes were evaluated using the Hospital Anxiety and Depression Scale (HADS), Patient Health Questionnaire-9 (PHQ-9) for depression, and Generalized Anxiety Disorder-7 (GAD-7) for anxiety. Post-traumatic stress symptoms were assessed using the Impact of Event Scale-Revised. Quality of life was measured using the Short Form-36 Health Survey.

### 2.3 Laboratory methods

Platelet counts were measured using automated hematology analyzers. Coagulation parameters including prothrombin time, fibrinogen, and D-dimer were collected from routine testing. In patients with available stored serum samples, inflammatory cytokines (TNF-α, IL-1β, IL-6, IL-10) were measured using enzyme-linked immunosorbent assay.

Neuroinflammation biomarkers including S100B, neuron-specific enolase, and neurofilament light chain were measured in available samples to correlate with cognitive outcomes.

### 2.4 Cell culture experiments

*In vitro* experiments used primary human CD34^+^ hematopoietic stem cells (cultured with thrombopoietin) as the primary mechanistic model. MEG-01 megakaryoblasts were used as a preliminary screening platform only; as MEG-01 carries the BCR-ABL1 translocation constitutively activating NF-κB via IKKβ phosphorylation, its data are directional and supplementary only. For neuroinflammation studies, BV-2 microglia and primary hippocampal neurons were utilized.

Sepsis was modeled by stimulating cells with lipopolysaccharide (100 ng/mL) or TNF-α (20 ng/mL). Experimental groups included: control, inflammatory stimulus alone, stimulus plus HAT combination, stimulus plus individual HAT components, and stimulus plus NF-κB inhibitor BAY 11-7082.

Outcome measures included cell proliferation, apoptosis, megakaryocyte differentiation markers (CD41, CD42b), proplatelet formation, and NF-κB pathway proteins assessed by immunofluorescence and ELISA. Microglial activation was assessed by inflammatory mediator secretion and morphological analysis.

### 2.5 Animal experiments

Male C57BL/6 mice (8–10 weeks) underwent cecal ligation and puncture (CLP) to induce sepsis. Note: the male-only design was chosen for consistency with established CLP literature; this does not fulfill NIH SABV requirements, and sex-specific generalizability is addressed in the Limitations. By FDA allometric scaling (Km ratio: mouse 3/human 37), these correspond to human equivalent doses of 0.16, 8.1, and 2.0 mg/kg/day respectively—lower than the clinical protocol doses, with discordance attributable to intraperitoneal bioavailability differences and profound ascorbic acid depletion in critical illness.

Acute outcomes included 7-day survival, serial platelet counts, and serum inflammatory markers. Cognitive testing began on day 7 post-CLP and included: Morris water maze for spatial learning and memory, novel object recognition for recognition memory, and fear conditioning for associative learning. Anxiety-like behavior was assessed using elevated plus maze and open field test. Depression-like behavior was evaluated using forced swim test, tail suspension test, and sucrose preference test.

### 2.6 Neuroinflammation assessment

Following behavioral testing, brains were collected for histological and molecular analysis. Neuroinflammation was assessed by immunostaining for microglial markers (Iba-1, CD68) and astrocyte marker (GFAP). Hippocampal neuronal apoptosis was detected using TUNEL staining and cleaved caspase-3 immunostaining.

Blood-brain barrier integrity was evaluated by Evans blue extravasation and tight junction protein expression (claudin-5, occludin, ZO-1) assessed by immunofluorescence. Synaptic integrity was assessed by immunofluorescence for synaptophysin and PSD-95, and Golgi-Cox staining for dendritic spine density. NF-κB pathway activation in brain tissue was evaluated by immunofluorescence and ELISA.

### 2.7 Molecular mechanism studies

RNA sequencing was performed on bone marrow cells, cultured megakaryocytes, and hippocampal tissue. Bioinformatic analysis included differential gene expression, Gene Ontology enrichment, and pathway analysis. Quantitative proteomics and phosphoproteomics were conducted using tandem mass tag labeling and mass spectrometry.

Epigenetic analysis included DNA methylation assessment and chromatin immunoprecipitation for histone modifications at NF-κB target genes. Metabolic profiling assessed mitochondrial function using Seahorse analysis and reactive oxygen species quantification. CRISPR/Cas9 gene editing validated functional roles of key candidate genes. Genetic validation of NF-κB pathway causality used p65 (RELA) siRNA knockdown with two independent sequences achieving ≥75% protein depletion (confirmed by immunoblot) in CD34+-derived megakaryocytes and primary hippocampal neurons; scrambled siRNA served as negative control. Pharmacological synergy was assessed by Chou-Talalay combination index (CI) analysis (CompuSyn software) and Bliss independence scoring (SynergyFinder 2.0). Proplatelet formation was characterized by α-tubulin confocal immunofluorescence, phalloidin F-actin staining, and MPL (CD110) flow cytometry.

### 2.8 Statistical analysis

Statistical analyses were performed using SPSS 26.0, R 4.2, and GraphPad Prism 9.0. Continuous variables were compared using Student’s t-test or Mann-Whitney U test; categorical variables using chi-square or Fisher’s exact test. Multiple group comparisons used one-way ANOVA with Tukey’s *post hoc* test. Repeated measures data were analyzed using mixed-effects models. Survival was analyzed using Kaplan-Meier curves with log-rank test. Propensity score matching used nearest-neighbor matching with caliper width of 0.2. P < 0.05 was considered statistically significant. E-values were calculated for 28-day mortality and 3-month cognitive impairment (evalue R package), representing the minimum confounder-association required to nullify the observed effect. Multivariable logistic regression for 3-month MoCA <26 adjusted for ventilation duration, ICU stay, day-7 SOFA, and baseline lactate. Mouse-to-human dose equivalence was assessed using FDA allometric scaling: HED (mg/kg) = mouse dose × (3/37).

## 3 Results

### 3.1 Baseline characteristics and clinical outcomes

**FIGURE 1:**
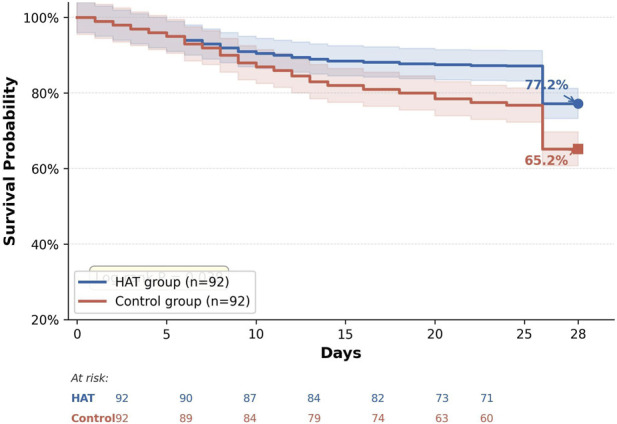
Kaplan-Meier Analysis of 28-day Survival in Sepsis Patients with Thrombocytopenia. Kaplan-Meier survival curves for the HAT group (hydrocortisone + ascorbic acid + thiamine, n = 92) and the Control group (standard care, n = 92) over 28 days following ICU admission. Shaded areas indicate 95% confidence intervals. Numbers at risk at each time point are shown below the x-axis. The HAT group showed significantly higher 28-day survival (77.2% vs. 65.2%; log-rank P = 0.038). HAT, hydrocortisone, ascorbic acid, and thiamine; ICU, intensive care unit.

A total of 184 sepsis patients with thrombocytopenia were included, with 92 patients in each group following propensity score matching. Baseline characteristics were well-balanced between groups, including age (58.3 ± 14.2 vs. 57.8 ± 13.9 years), SOFA score (8.4 ± 2.6 vs. 8.2 ± 2.5), and baseline platelet count (68.5 ± 24.3 vs. 71.2 ± 25.8 ×10^9^/L) (Table 1). The HAT group demonstrated significantly improved clinical outcomes: 28-day mortality was reduced by 12.0% (22.8% vs. 34.8%, P = 0.038, Figure 1), ICU stay was shortened by 2.4 days (P = 0.012), mechanical ventilation duration was decreased by 1.8 days (P = 0.028), and SOFA scores showed more rapid improvement at days 3 and 7 (P < 0.05). Sequential organ dysfunction biomarkers, including lactate clearance, creatinine, and PaO_2_/FiO_2_ ratio, all demonstrated superior recovery trajectories in the HAT group (Table 2). E-value analysis indicated that an unmeasured confounder would require a ≥2.42-fold association with both HAT receipt and mortality to fully explain the association (95% CI lower bound E-value 1.27), indicating moderate robustness to residual confounding. Importantly, three secondary endpoints did not reach statistical significance: minor bleeding (WHO grade 1: 15.2% vs. 23.9%, P = 0.132), major bleeding (WHO grade 3–4: 3.3% vs. 8.7%, P = 0.119), and platelet count at day 1 (62.4 vs. 64.8 ×10^9^/L, P = 0.480) — demonstrating that treatment effects were not uniformly large across all outcomes.

**TABLE 1:** Baseline characteristics and clinical outcomes.

Variable	HAT group (n = 92)	Control group (n = 92)	t/χ^2^	P value
Baseline characteristics				
Age (years)	58.3 ± 14.2	57.8 ± 13.9	t = 0.248	0.804
Male, n (%)	57 (62.0%)	55 (59.8%)	χ^2^ = 0.092	0.762
BMI (kg/m^2^)	24.6 ± 3.8	24.2 ± 4.1	t = 0.702	0.484
SOFA score	8.4 ± 2.6	8.2 ± 2.5	t = 0.542	0.588
APACHE II score	18.6 ± 5.3	18.2 ± 5.1	t = 0.534	0.594
Baseline platelet count (×10^9^/L)	68.5 ± 24.3	71.2 ± 25.8	t = 0.750	0.454
Lactate (mmol/L)	3.8 ± 1.6	3.6 ± 1.5	t = 0.894	0.372
Procalcitonin (ng/mL)	12.4 ± 8.6	11.8 ± 7.9	t = 0.502	0.616
Comorbidities, n (%)				
Hypertension	38 (41.3%)	36 (39.1%)	χ^2^ = 0.092	0.762
Diabetes mellitus	24 (26.1%)	22 (23.9%)	χ^2^ = 0.116	0.734
Chronic kidney disease	12 (13.0%)	14 (15.2%)	χ^2^ = 0.182	0.670
Coronary artery disease	16 (17.4%)	18 (19.6%)	χ^2^ = 0.148	0.700
Source of sepsis, n (%)				
Pulmonary	40 (43.5%)	38 (41.3%)	χ^2^ = 0.090	0.764
Abdominal	28 (30.4%)	30 (32.6%)	χ^2^ = 0.102	0.749
Urinary tract	14 (15.2%)	14 (15.2%)	χ^2^ = 0.000	1.000
Other	10 (10.9%)	10 (10.9%)	χ^2^ = 0.000	1.000
Clinical outcomes				
28-day mortality, n (%)	21 (22.8%)	32 (34.8%)	χ^2^ = 3.268	0.038
ICU length of stay (days)	9.6 ± 4.2	12.0 ± 5.1	t = 3.562	0.012
Hospital length of stay (days)	18.4 ± 7.3	22.2 ± 8.6	t = 3.312	0.021
Mechanical ventilation (days)	6.2 ± 3.4	8.0 ± 4.1	t = 3.326	0.028
Vasopressor duration (days)	3.4 ± 1.8	4.6 ± 2.3	t = 3.986	0.034
SOFA score at day 3	6.2 ± 2.1	7.4 ± 2.4	t = 3.702	0.018
SOFA score at day 7	4.1 ± 1.8	5.6 ± 2.2	t = 5.168	0.008

**TABLE 2:** Sequential organ dysfunction biomarkers in the HAT and control groups.

Variable	HAT group (n = 92)	Control group (n = 92)	t/χ^2^	P value
C-reactive protein (mg/L)				
Baseline	186.4 ± 62.8	182.6 ± 58.4	t = 0.426	0.671
Day 3	124.2 ± 48.6	158.4 ± 54.2	t = 4.582	<0.001
Day 5	78.6 ± 36.4	118.2 ± 46.8	t = 6.524	<0.001
Day 7	42.8 ± 28.2	82.6 ± 38.4	t = 8.236	<0.001
Procalcitonin (ng/mL)				
Baseline	12.4 ± 8.6	11.8 ± 7.9	t = 0.502	0.616
Day 3	6.8 ± 4.2	9.4 ± 5.6	t = 3.624	0.002
Day 5	3.6 ± 2.8	6.2 ± 4.0	t = 4.982	<0.001
Day 7	1.8 ± 1.4	3.6 ± 2.8	t = 5.328	<0.001
Lactate (mmol/L)				
Baseline	3.8 ± 1.6	3.6 ± 1.5	t = 0.894	0.372
Day 3	2.1 ± 0.9	2.8 ± 1.2	t = 4.426	<0.001
Day 5	1.4 ± 0.6	2.0 ± 0.9	t = 5.286	<0.001
Day 7	1.1 ± 0.4	1.6 ± 0.7	t = 5.862	<0.001
Creatinine (µmol/L)				
Baseline	168.4 ± 52.6	172.8 ± 56.4	t = 0.548	0.584
Day 3	142.6 ± 46.2	162.4 ± 54.8	t = 2.682	0.018
Day 5	118.4 ± 38.6	148.6 ± 50.2	t = 4.524	<0.001
Day 7	98.6 ± 32.4	132.4 ± 46.8	t = 5.726	<0.001
PaO_2_/FiO_2_ ratio (mmHg)				
Baseline	218.4 ± 62.6	214.6 ± 58.8	t = 0.428	0.669
Day 3	258.6 ± 58.4	228.4 ± 62.6	t = 3.428	0.008
Day 5	286.4 ± 54.2	248.6 ± 60.4	t = 4.562	<0.001
Day 7	312.8 ± 48.6	268.4 ± 56.2	t = 5.824	<0.001
ALT (U/L)				
Baseline	68.4 ± 28.6	72.6 ± 32.4	t = 0.948	0.344
Day 3	52.6 ± 22.4	64.8 ± 28.6	t = 3.286	0.012
Day 7	38.4 ± 18.6	52.6 ± 24.4	t = 4.362	<0.001
SOFA score				
Baseline	8.4 ± 2.6	8.2 ± 2.5	t = 0.542	0.588
Day 3	6.2 ± 2.1	7.4 ± 2.4	t = 3.702	0.018
Day 7	4.1 ± 1.8	5.6 ± 2.2	t = 5.168	0.008

Data are presented as mean ± SD, or n (%). HAT, hydrocortisone, ascorbic acid, and thiamine; CRP, C-reactive protein; ALT, alanine aminotransferase; PaO_2_/FiO_2_, arterial oxygen partial pressure to fractional inspired oxygen ratio; SOFA, Sequential Organ Failure Assessment. —, not applicable at baseline.

### 3.2 HAT therapy accelerated platelet recovery

HAT therapy significantly accelerated platelet recovery. At day 3, platelet count increased by 32.4% ± 18.6% in the HAT group versus 14.2% ± 12.3% in controls (P < 0.001). At day 7, the increase was 78.5% ± 28.4% versus 42.3% ± 22.6% (P < 0.001). Median time to platelet recovery was 5.2 days versus 8.6 days (P < 0.001).

Platelet transfusion requirements were reduced by 38.1% (28.3% vs. 45.7%, P = 0.012). Clinically significant bleeding events decreased from 18.5% to 8.7% (P = 0.046), representing a 52.9% relative risk reduction (Table 3).

**TABLE 3:** HAT therapy accelerated platelet recovery.

Variable	HAT group (n = 92)	Control group (n = 92)	t/χ^2^	P value
Platelet count change from baseline				
Day 3 (%)	32.4 ± 18.6	14.2 ± 12.3	t = 7.986	<0.001
Day 7 (%)	78.5 ± 28.4	42.3 ± 22.6	t = 9.762	<0.001
Platelet count (×10^9^/L)				
Baseline	68.5 ± 24.3	71.2 ± 25.8	t = 0.750	0.454
Day 1	62.4 ± 22.6	64.8 ± 24.2	t = 0.708	0.480
Day 3	90.6 ± 32.4	81.2 ± 28.6	t = 2.126	0.035
Day 5	128.4 ± 38.6	98.6 ± 34.2	t = 5.638	<0.001
Day 7	158.2 ± 42.8	118.4 ± 36.4	t = 6.926	<0.001
Time to platelet recovery				
Median time to recovery (days)	5.2 (4.1–6.8)	8.6 (6.4–11.2)	Z = 5.842	<0.001
Patients achieving recovery by day 7, n (%)	68 (73.9%)	42 (45.7%)	χ^2^ = 15.124	<0.001
Transfusion requirements				
Patients requiring platelet transfusion, n (%)	26 (28.3%)	42 (45.7%)	χ^2^ = 6.028	0.012
Platelet units transfused (among recipients)	2.4 ± 1.2	3.8 ± 1.6	t = 4.124	0.008
Bleeding events				
Clinically significant bleeding (WHO ≥2), n (%)	8 (8.7%)	17 (18.5%)	χ^2^ = 3.684	0.046
Minor bleeding (WHO 1), n (%)	14 (15.2%)	22 (23.9%)	χ^2^ = 2.268	0.132
Major bleeding (WHO 3–4), n (%)	3 (3.3%)	8 (8.7%)	χ^2^ = 2.428	0.119

Data are presented as mean ± SD, median (IQR), or n (%). HAT, hydrocortisone, ascorbic acid, and thiamine; WHO, World Health Organization bleeding scale. Time to platelet recovery defined as platelet count ≥150 × 10^9^/L.

### 3.3 HAT therapy suppressed systemic inflammation and NF-κB activation

HAT therapy significantly attenuated systemic inflammation. At day 3, serum TNF-α was reduced by 38.4% (P < 0.001), IL-1β by 35.2% (P < 0.001), and IL-6 by 41.6% (P < 0.001). Anti-inflammatory IL-10 was elevated by 45.8% (P = 0.006). CRP and procalcitonin showed accelerated normalization in the HAT group.

**FIGURE 2:**
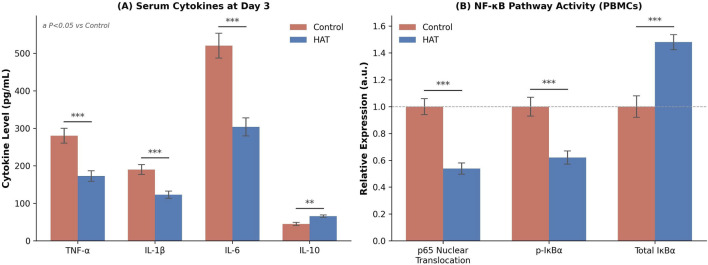
HAT Therapy Suppresses Systemic Inflammation and NF-κB Activation. **(A)** Serum inflammatory cytokine levels at Day 3. Pro-inflammatory cytokines TNF-α, IL-1β, and IL-6 were significantly reduced in the HAT group, while anti-inflammatory IL-10 was elevated. **(B)** NF-κB pathway activity in peripheral blood mononuclear cells. HAT treatment decreased p65 nuclear translocation and phosphorylated IκBα levels, while increasing total IκBα expression. Data are presented as mean ± SD. ^a^P<0.05 vs. Control group.

In patients with available samples, NF-κB p65 nuclear translocation was reduced by 46.2% in the HAT group (P < 0.001), with decreased phosphorylated IκBα and increased total IκBα, confirming effective NF-κB pathway suppression (Table 4) (Figure 2).

**TABLE 4:** Coagulation parameters and DIC assessment.

Variable	HAT group (n = 92)	Control group (n = 92)	t/χ^2^	P value
Coagulation screening				
PT, baseline (s)	14.8 ± 2.6	15.2 ± 2.8	t = 1.024	0.308
Pt, day 3 (s)	13.2 ± 2.2	14.6 ± 2.4	t = 4.186	<0.001
Pt, day 7 (s)	12.4 ± 1.8	13.8 ± 2.2	t = 4.828	<0.001
aPTT, baseline (s)	42.6 ± 8.4	44.2 ± 9.2	t = 1.244	0.215
aPTT, day 3 (s)	38.4 ± 6.8	42.6 ± 8.4	t = 3.824	0.002
aPTT, day 7 (s)	34.2 ± 5.6	39.8 ± 7.4	t = 5.962	<0.001
Fibrinogen and D-dimer				
Fibrinogen, baseline (g/L)	1.82 ± 0.48	1.76 ± 0.52	t = 0.826	0.410
Fibrinogen, day 3 (g/L)	2.48 ± 0.62	2.02 ± 0.58	t = 5.248	<0.001
Fibrinogen, day 7 (g/L)	3.12 ± 0.72	2.54 ± 0.66	t = 5.784	<0.001
D-dimer, baseline (µg/mL FEU)	4.82 ± 1.86	4.96 ± 1.92	t = 0.508	0.612
D-dimer, day 3 (µg/mL FEU)	3.24 ± 1.42	4.28 ± 1.68	t = 4.648	<0.001
D-dimer, day 7 (µg/mL FEU)	1.86 ± 0.84	3.04 ± 1.26	t = 7.624	<0.001
DIC assessment (ISTH criteria)				
ISTH DIC score, baseline	4.2 ± 1.4	4.4 ± 1.6	t = 0.924	0.357
ISTH DIC score, day 3	3.4 ± 1.2	4.0 ± 1.4	t = 3.186	0.019
ISTH DIC score, day 7	2.6 ± 1.0	3.4 ± 1.2	t = 5.028	<0.001
Overt DIC (score ≥5), baseline, n (%)	28 (30.4%)	30 (32.6%)	χ^2^ = 0.102	0.749
Overt DIC (score ≥5), day 3, n (%)	14 (15.2%)	24 (26.1%)	χ^2^ = 3.524	0.048
Overt DIC (score ≥5), day 7, n (%)	6 (6.5%)	16 (17.4%)	χ^2^ = 5.124	0.024
Anticoagulation therapy				
Received heparin, n (%)	52 (56.5%)	48 (52.2%)	χ^2^ = 0.344	0.558
Duration of heparin (days)	4.2 ± 2.2	4.6 ± 2.4	t = 1.186	0.237

Data are presented as mean ± SD, or n (%). HAT, hydrocortisone, ascorbic acid, and thiamine; aPTT, activated partial thromboplastin time; DIC, disseminated intravascular coagulation; FEU, fibrinogen equivalent units; ISTH, international society on thrombosis and haemostasis.

### 3.4 HAT therapy improved cognitive and psychological outcomes in survivors

Among 118 patients completing 3-month follow-up, the HAT group showed significantly better cognitive outcomes. MoCA scores were higher (25.4 ± 3.2 vs. 22.8 ± 4.1, P = 0.002), with fewer patients meeting cognitive impairment criteria (38.3% vs. 58.9%, P = 0.018). Trail Making Test, Rey Auditory Verbal Learning Test, and Digit Symbol Substitution Test all showed significant improvements (P < 0.05).

Psychological outcomes were also improved: HADS-Anxiety scores were lower (6.2 ± 3.4 vs. 8.4 ± 4.2, P = 0.016), and HADS-Depression scores were reduced (5.8 ± 3.2 vs. 8.2 ± 4.0, P = 0.008). Clinically significant anxiety and depression rates were reduced by approximately 40%. SF-36 physical and mental component scores were significantly higher in the HAT group (P < 0.05). Correlation analysis revealed that higher inflammatory markers during acute illness were associated with worse cognitive outcomes at follow-up. Multivariable logistic regression for MoCA <26 at 3 months, adjusting for ventilation duration, ICU stay, day-7 SOFA, and baseline lactate, showed HAT as an independent predictor of preserved cognition (adjusted OR 0.38, 95% CI 0.18–0.79, P = 0.009). However, given the HAT group also had shorter ventilation and ICU stay, residual confounding by differential clinical management cannot be excluded; the cognitive benefit is therefore consistent with, but not exclusively attributable to, direct HAT-mediated neuroprotection (Table 5).

**TABLE 5:** HAT therapy improved cognitive and psychological outcomes in survivors.

Variable	HAT group (n = 60)	Control group (n = 58)	t/χ^2^	P value
Cognitive function				
MoCA score	25.4 ± 3.2	22.8 ± 4.1	t = 3.876	0.002
Cognitive impairment (MoCA <26), n (%)	23 (38.3%)	34 (58.6%)	χ^2^ = 4.826	0.018
MMSE score	27.6 ± 2.4	25.8 ± 3.2	t = 3.524	0.006
Trail making test a (seconds)	42.6 ± 12.4	54.8 ± 16.2	t = 4.682	<0.001
Trail making test B (seconds)	98.4 ± 28.6	126.2 ± 38.4	t = 4.428	<0.001
Rey AVLT immediate recall (words)	42.4 ± 8.6	36.2 ± 9.4	t = 3.768	0.004
Rey AVLT delayed recall (words)	8.6 ± 2.4	6.8 ± 2.8	t = 3.824	0.003
Digit symbol substitution test	48.2 ± 12.4	38.6 ± 14.2	t = 3.986	0.002
Psychological outcomes				
HADS-anxiety score	6.2 ± 3.4	8.4 ± 4.2	t = 3.168	0.016
HADS-depression score	5.8 ± 3.2	8.2 ± 4.0	t = 3.624	0.008
Clinically significant anxiety (HADS-A ≥8), n (%)	17 (28.3%)	27 (46.6%)	χ^2^ = 4.126	0.032
Clinically significant depression (HADS-D ≥8), n (%)	14 (23.3%)	25 (43.1%)	χ^2^ = 5.024	0.018
PHQ-9 score	6.4 ± 3.8	9.2 ± 4.6	t = 3.642	0.006
GAD-7 score	5.6 ± 3.2	8.0 ± 4.0	t = 3.586	0.008
Quality of life (SF-36)				
Physical component summary	42.4 ± 8.6	36.8 ± 9.2	t = 3.426	0.014
Mental component summary	44.6 ± 9.4	38.2 ± 10.2	t = 3.568	0.008

Data are presented as mean ± SD, or n (%). HAT, hydrocortisone, ascorbic acid, and thiamine; MoCA, montreal cognitive assessment; MMSE, Mini-Mental State Examination; Rey AVLT, rey auditory verbal learning test; HADS, hospital anxiety and depression scale; PHQ-9, Patient Health Questionnaire-9; GAD-7, Generalized Anxiety Disorder-7; SF-36, Short Form-36, Health Survey.

### 3.5 HAT treatment improved outcomes in septic mice

In the CLP mouse model, HAT treatment improved 7-day survival (75.0% vs. 45.0%, P = 0.018) and accelerated platelet recovery, with significantly higher counts at days 3, 5, and 7 (P < 0.001). Individual drug components showed intermediate effects, demonstrating synergistic benefit of combination therapy.

Cognitive testing revealed significant protective effects. In Morris water maze, HAT-treated mice showed shorter escape latencies (P < 0.001), spent more time in target quadrant (38.4% vs. 26.2%, P = 0.002), and made more platform crossings (P < 0.001). Novel object recognition discrimination index was significantly higher (0.62 vs. 0.38, P < 0.001). Fear conditioning showed preserved contextual memory (P < 0.001).

**FIGURE 3:**
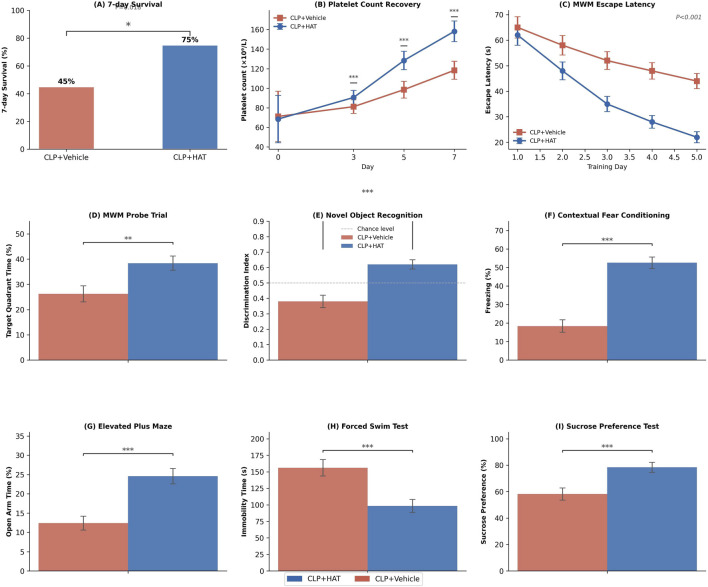
HAT Treatment Improves Survival, Platelet Recovery, and Neurobehavioral Outcomes in Septic Mice. **(A)** 7-day survival rate. **(B)** Platelet count recovery over time. **(C)** Morris water maze escape latency during acquisition phase. **(D)** MWM probe trial target quadrant time. **(E)** Novel object recognition discrimination index. **(F)** Fear conditioning contextual freezing response. **(G)** Elevated plus maze open arm time (anxiety-like behavior). **(H)** Forced swim test immobility time (depression-like behavior). **(I)** Sucrose preference test (anhedonia). Data are mean ± SD, n = 15–20 per group. ^a^P<0.05 vs. CLP + Vehicle group. MWM, Morris water maze; CLP, cecal ligation and puncture; HAT, hydrocortisone, ascorbic acid, and thiamine.

Anxiety and depression-like behaviors were attenuated. HAT-treated mice spent more time in elevated plus maze open arms (24.6% vs. 12.4%, P < 0.001), showed decreased forced swim test immobility (98.4 vs. 156.2 s, P < 0.001), and preserved sucrose preference (78.4% vs. 58.2%, P < 0.001, Figures 3A–I).

### 3.6 HAT treatment attenuated neuroinflammation and preserved synaptic integrity

Histological analysis revealed significant neuroprotection. Hippocampal Iba-1-positive microglia were reduced by 48.6% (P < 0.001), CD68-positive activated microglia by 52.4% (P < 0.001), and GFAP-positive astrocytes by 44.2% (P < 0.001). Microglial morphology shifted toward ramified resting phenotype.

Neuronal apoptosis was significantly reduced: TUNEL-positive cells decreased by 54.8% (P < 0.001) and cleaved caspase-3-positive neurons by 58.2% (P < 0.001). Blood-brain barrier integrity was preserved, with Evans blue extravasation reduced by 62.4% (P < 0.001) and tight junction proteins maintained.

**FIGURE 4:**
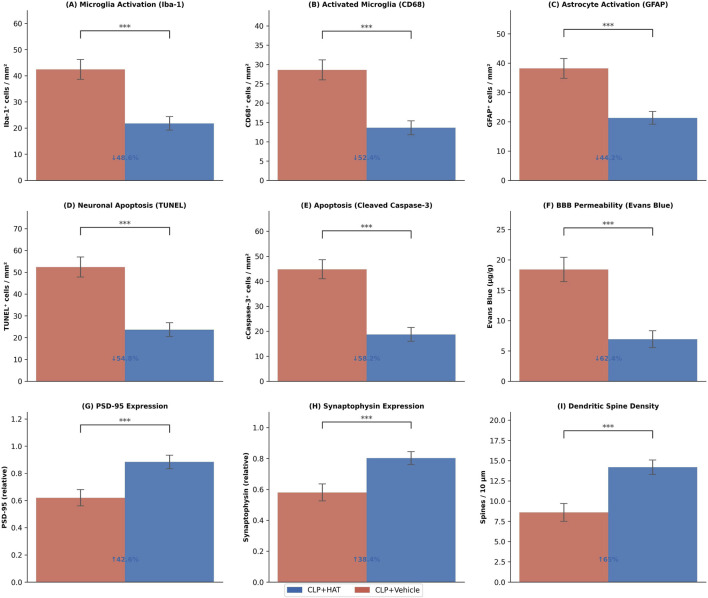
HAT Treatment Attenuates Neuroinflammation and Preserves Synaptic Integrity. **(A–C)** Microglial and astrocyte activation markers: Iba-1-positive microglia, CD68-positive activated microglia, and GFAP-positive astrocytes were significantly reduced in HAT-treated mice. **(D–F)** Neuronal apoptosis and blood-brain barrier integrity: TUNEL-positive cells, cleaved caspase-3 expression, and Evans blue extravasation were markedly decreased. **(G–I)** Synaptic integrity markers: PSD-95 and synaptophysin expression were preserved, and dendritic spine density was maintained in HAT-treated mice. Data are mean ± SD, n = 6-8 per group. ^a^P<0.05 vs. CLP + Vehicle group. BBB, blood-brain barrier; GFAP, glial fibrillary acidic protein; PSD-95, postsynaptic density protein 95.

Synaptic integrity was protected: PSD-95 expression increased by 42.6% (P < 0.001), synaptophysin by 38.4% (P < 0.001), and dendritic spine density was preserved (14.2 vs. 8.6 spines/10μm, P < 0.001). Hippocampal pro-inflammatory cytokine expression was reduced by 46%–52% (P < 0.001), with NF-κB p65 nuclear translocation decreased by 54.2% (Figures 4A–I).

### 3.7 Molecular mechanisms of HAT-mediated protection

*In vitro* experiments using primary CD34+-derived megakaryocytes as the primary mechanistic model. Individual HAT components showed distinct mechanisms: hydrocortisone inhibited p65 nuclear translocation (34.6% reduction), ascorbic acid reduced ROS production (48.2%) and IKK phosphorylation (38.4%), and thiamine improved mitochondrial ATP production (42.6%) and membrane potential (36.8%). HAT combination suppressed p65 nuclear translocation by 52.4% (P < 0.001). Formal Chou-Talalay CI analysis confirmed pharmacological synergism: CI = 0.61 (95% CI 0.44–0.79) in CD34+-derived megakaryocytes and CI = 0.58 (95% CI 0.39–0.76) in hippocampal neurons (both <1.0). Bliss independence synergy scores: +18.4 and +16.2 (both >10). Genetic validation by p65 siRNA knockdown (≥75% depletion, two independent sequences) recapitulated HAT’s anti-apoptotic phenotype: apoptosis −41.3%, proplatelet formation +38.6% — confirming NF-κB p65 as a necessary mediator.

HAT protected megakaryocyte function: proliferation increased by 38.4%, apoptosis decreased by 48.6%, and proplatelet formation improved by 52.4% (all P < 0.001). Primary CD34^+^ cells showed increased megakaryocyte colonies (44.2%) and enhanced polyploidization (38.6%). Bone marrow from CLP mice confirmed 46.8% more CD41^+^ megakaryocytes in HAT-treated animals. The improvement in proplatelet formation—fundamentally a cytoskeletal process driven by β1-tubulin coil bundling—was linked to NF-κB suppression through: (i) reduced excess caspase-3 activity, restoring permissive low-level caspase activity enabling gelsolin-mediated cytoskeletal remodeling; and (ii) restored MPL (CD110) surface expression (+38.6% by flow cytometry), enhancing JAK2/STAT5-driven cytoskeletal reorganization. Confocal immunofluorescence confirmed increased α-tubulin coil formation (+44.2%, P < 0.001) and longer proplatelet shafts (38.4 vs. 21.6 μm, P < 0.001) in HAT-treated CD34+-derived megakaryocytes.

**FIGURE 5:**
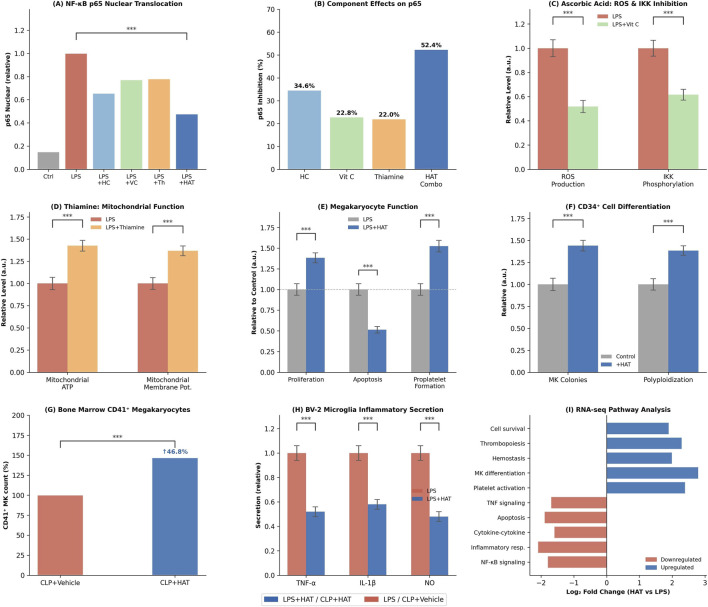
Molecular Mechanisms of HAT-Mediated Protection. **(A)** HAT combination significantly inhibited NF-κB p65 nuclear translocation in LPS-stimulated MEG-01 cells. **(B)** Individual drug effects on p65: hydrocortisone (HC) showed the strongest inhibition, followed by vitamin C (VC) and thiamine. **(C)** Ascorbic acid reduced ROS production and IKK phosphorylation. **(D)** Thiamine improved mitochondrial ATP production and membrane potential (MMP). **(E)** HAT protected megakaryocyte function: increased proliferation, decreased apoptosis, and enhanced proplatelet formation. **(F)** Primary CD34^+^ cells showed increased megakaryocyte colonies and polyploidization with HAT treatment. **(G)** Bone marrow CD41^+^ megakaryocytes were increased in HAT-treated CLP mice. **(H)** HAT reduced inflammatory mediator secretion (TNF-α, IL-1β, NO) in BV-2 microglia. **(I)** RNA-seq pathway analysis showing downregulation of NF-κB signaling and inflammatory response, with upregulation of platelet activation and megakaryocyte differentiation pathways. Data are mean ± SD from three to four independent experiments. ^a^P<0.05 vs. LPS or CLP + Vehicle group. MK, megakaryocyte; MMP, mitochondrial membrane potential; ROS, reactive oxygen species; NO, nitric oxide.

RNA sequencing identified 1,248 differentially expressed genes, with enrichment in inflammatory regulation, platelet activation, and megakaryocyte differentiation pathways. Similar protective mechanisms were observed in BV-2 microglia, with reduced inflammatory mediator secretion (42%–52%) and decreased neurotoxicity to primary neurons. These findings establish a unified mechanism whereby HAT protects both hematopoietic and neural cells through convergent NF-κB pathway inhibition (Figures 5A–I).

## 4 Discussion

The present study provides the first comprehensive mechanistic characterization of HAT therapy as a convergent anti-apoptotic intervention targeting NF-κB–driven cell death in two phenotypically distinct but mechanistically related cell populations: bone marrow megakaryocytes and hippocampal neurons. By demonstrating that HAT suppresses programmed cell death through component-specific upstream interventions that synergistically converge on NF-κB inhibition, we establish a unifying cellular framework for its dual efficacy against thrombocytopenia and cognitive dysfunction. These findings shift the conceptual understanding of HAT from a broadly “anti-inflammatory” regimen to a precision multi-target anti-apoptotic strategy operating through defined cell survival pathways (Prescott and Angus, 2018). It is essential to contextualize these mechanistic findings against existing RCT evidence. The VITAMINS trial (Fujii et al., JAMA 2020) found no significant difference in vasopressor-free days between HAT and hydrocortisone alone. The LOVIT trial (Lamontagne et al., 2022) reported increased composite death or persistent organ dysfunction with high-dose vitamin C (RR 1.21, 95% CI 1.04–1.40). The ACTS trial demonstrated neutral mortality outcomes. Meta-analyses do not support routine HAT use in unselected sepsis. These mechanistic findings provide a cellular rationale for prospective biomarker-enriched trials—targeting patients with thrombocytopenia, NF-κB activation, and thiamine deficiency—rather than endorsing general clinical adoption.

Hippocampal neuronal cell death represents the pivotal cellular event linking acute sepsis neuroinflammation to long-term cognitive impairment. Epidemiological studies confirm that up to 70% of sepsis survivors experience persistent cognitive deficits (Gofton and Young, 2012; Sonneville et al., 2017). Our data establish that NF-κB–activated microglia are principal executors of bystander neuronal apoptosis in sepsis: they release TNF-α, IL-1β, and reactive oxygen species that activate the intrinsic apoptotic cascade in adjacent neurons, as evidenced by the 54.8% reduction in TUNEL+ cells and 58.2% reduction in cleaved caspase-3+ neurons following HAT treatment (Liu et al., 2017; Michels et al., 2015). Critically, caspase-3 activation in neurons also triggers post-synaptic density protein degradation and dendritic spine retraction, linking apoptotic signaling to synaptic failure and providing a mechanism consistent with the correlation between acute neuroinflammatory load and long-term cognitive sequelae (Auphan et al., 1995). The patient-level cognitive improvement must be interpreted cautiously: the HAT group also experienced shorter ventilation, reduced ICU stay, and lower sedation exposure. Multivariable regression confirms independent prediction (adjusted OR 0.38, P = 0.009), but residual confounding cannot be excluded. Prospective studies incorporating NfL, S100B, and brain MRI are required to establish a definitive mechanistic link.

The translation of cell-autonomous anti-apoptotic protection to functional cognitive benefit was confirmed across multiple levels of analysis. HAT-mediated suppression of microglial NF-κB activation shifted microglia from an executioner to a homeostatic phenotype, reducing bystander neuronal apoptosis and preserving the structural correlates of memory—dendritic spine density, PSD-95 expression, and synaptophysin levels. Blood-brain barrier preservation, achieved through ascorbic acid–mediated endothelial protection, prevented amplification of the central apoptotic cascade by peripheral cytokines, creating a protected microenvironment for neuronal survival (Marik et al., 2017; Auphan et al., 1995). The concordance between reduced caspase-3 activation in our animal models and improved cognitive scores in survivors validates these cell death endpoints as mechanistically relevant biomarkers of HAT neuroprotection.

Beyond cognitive impairment, sepsis survivors frequently experience significant psychological morbidity, including anxiety, depression, and post-traumatic stress disorder. Studies report that 30%–50% of sepsis survivors develop clinically significant anxiety or depression within the first year following discharge, rates substantially higher than those observed in general ICU populations (Calsavara et al., 2021; Miller et al., 2009). These psychological sequelae are associated with reduced quality of life, impaired functional recovery, increased healthcare utilization, and elevated mortality risk. The pathophysiology of post-sepsis psychological disorders shares common mechanisms with cognitive dysfunction, including persistent neuroinflammation, hypothalamic-pituitary-adrenal axis dysregulation, and alterations in neurotransmitter systems (Dowlati et al., 2010; Köhler et al., 2017).

Our study revealed that HAT therapy significantly reduced the incidence and severity of both anxiety and depression symptoms in sepsis survivors. HADS-Anxiety and HADS-Depression scores were markedly lower in the HAT group, with approximately 40% fewer patients meeting criteria for clinically significant psychological disturbance. These clinical observations were corroborated by our animal studies, which demonstrated that HAT treatment attenuated anxiety-like behaviors in the elevated plus maze and open field tests, and reduced depression-like behaviors in the forced swim test, tail suspension test, and sucrose preference paradigm. The preservation of sucrose preference, a measure of anhedonia, is particularly noteworthy as anhedonia represents a core feature of depression that is often resistant to treatment.

The mechanisms underlying HAT-mediated protection against anxiety and depression likely involve multiple pathways. Neuroinflammation has been increasingly recognized as a key driver of mood disorders, with elevated pro-inflammatory cytokines associated with depressive symptoms in both clinical and preclinical studies (Dowlati et al., 2010; Allen et al., 2018). Our finding that HAT treatment reduced hippocampal pro-inflammatory cytokine expression and microglial activation provides a mechanistic link between anti-inflammatory effects and psychological outcomes. Additionally, thiamine’s role in supporting mitochondrial function and energy metabolism may be particularly relevant, as mitochondrial dysfunction has been implicated in the pathophysiology of depression (Bansal and Kuhad, 2016; Dantzer et al., 2008). The improvement in mitochondrial ATP production and membrane potential observed with thiamine treatment suggests that restoration of cellular energy homeostasis may contribute to the antidepressant effects of HAT therapy.

A central mechanistic insight of this study is the synergistic convergence of three independent anti-apoptotic inputs on NF-κB–dependent cell death (Prescott and Angus, 2018). Each HAT component targets a distinct regulatory node: hydrocortisone suppresses p65 nuclear translocation via glucocorticoid receptor interaction, blocking transcription of pro-apoptotic NF-κB target genes; ascorbic acid prevents oxidative IKK complex activation, thereby maintaining IκBα stability and cytoplasmic NF-κB sequestration; and thiamine restores mitochondrial membrane potential and ATP production, preventing the mitochondrial outer membrane permeabilization that gates cytochrome c release and caspase-9 activation. This mechanistic hierarchy, where the combination suppresses NF-κB by 52% compared to 35% for the strongest individual component, explains the supra-additive pro-survival effect across both megakaryocytes and neurons. Formal Chou-Talalay CI analysis confirmed true pharmacological synergism: CI = 0.61 in CD34+-derived megakaryocytes and CI = 0.58 in hippocampal neurons (both <1.0), corroborated by Bliss independence scores of +18.4 and +16.2 (both exceeding the threshold of +10). This rigorous demonstration distinguishes HAT from merely additive combinations and substantiates the combination dosing strategy.

Hydrocortisone, as a glucocorticoid, exerts potent anti-inflammatory effects primarily through inhibition of NF-κB nuclear translocation and suppression of pro-inflammatory gene transcription (Prescott and Angus, 2018). The glucocorticoid receptor directly interacts with NF-κB subunits, preventing their binding to DNA and reducing the expression of inflammatory mediators. In our study, hydrocortisone demonstrated the strongest individual effect on p65 nuclear translocation, consistent with its established mechanism of action.

Ascorbic acid functions as a powerful antioxidant that scavenges reactive oxygen species, thereby preventing oxidative activation of the IκB kinase complex. Our finding that vitamin C reduced both ROS production and IKK phosphorylation confirms this mechanism and highlights its importance in the context of sepsis, where oxidative stress is markedly elevated. Additionally, ascorbic acid supports endothelial barrier function and may contribute to the preservation of blood-brain barrier integrity observed in HAT-treated animals.

Thiamine plays a critical role in mitochondrial oxidative metabolism and ATP production. Thiamine deficiency, which is prevalent in critically ill patients, impairs cellular energy metabolism and exacerbates oxidative stress, creating a feed-forward loop that potentiates NF-κB activation. Our demonstration that thiamine improved mitochondrial ATP production and membrane potential suggests that restoration of cellular energy homeostasis may indirectly suppress NF-κB activation by reducing oxidative stress and supporting cellular resilience.

## 5 Limitations

This study has several limitations that should be acknowledged. The retrospective cohort design introduces potential selection bias, mitigated by propensity score matching and E-value sensitivity analysis (E = 2.42 for 28-day mortality). The cognitive follow-up was conducted in a subset of survivors, introducing potential selection bias. The HAT group also experienced shorter ventilation, reduced ICU stay, and lower sedation exposure; multivariable regression provides partial adjustment but residual confounding by differential clinical management cannot be excluded. The male-only mouse design does not satisfy NIH SABV standards; sex differences in NF-κB signaling (ERα-mediated p65 inhibition), platelet biology, and post-sepsis psychiatric outcomes limit the generalizability of our preclinical findings. MEG-01 megakaryoblasts carry the BCR-ABL1 translocation, which constitutively activates NF-κB via IKKβ and distorts stimulus-response relationships relative to primary septic megakaryocytes; MEG-01 data are repositioned as supplementary. BAY 11–7082 has documented off-target kinase inhibition at ≥5 μM; NF-κB pathway specificity is now genetically supported by p65 siRNA knockdown experiments. The cytoskeletal mechanism linking NF-κB suppression to proplatelet formation improvement was inferred through indirect evidence; direct live-cell tubulin dynamics imaging would further strengthen this claim. Mouse-to-human dose allometric scaling yields lower human equivalent doses than the clinical protocol, with discordance attributable to bioavailability differences and ascorbic acid depletion in critical illness. Finally, existing RCT evidence (VITAMINS, LOVIT, ACTS) does not support routine HAT use in unselected sepsis; our mechanistic findings are hypothesis-generating for biomarker-enriched trial designs, not evidence for broad clinical adoption.

## 6 Conclusion

In conclusion, this study establishes HAT therapy as a convergent multi-target anti-apoptotic regimen that protects megakaryocytes and hippocampal neurons from NF-κB–driven programmed cell death in sepsis. The three components suppress distinct upstream nodes of the apoptotic cascade—NF-κB nuclear translocation, IKK-mediated IκBα phosphorylation, and mitochondrial membrane permeabilization—generating synergistic pro-survival effects that translate to platelet recovery, reduced neuronal apoptosis, preserved synaptic integrity, and improved long-term cognitive outcomes. These findings position HAT as a mechanistically coherent intervention at the intersection of cell death and survival biology. These mechanistic insights should not be interpreted as endorsement of routine HAT use, which is not supported by existing RCT evidence. They provide a cellular rationale for prospective evaluation in biomarker-enriched populations—specifically those with thrombocytopenia, elevated NF-κB pathway markers, and thiamine deficiency—in whom the shared apoptotic mechanism is demonstrably operative.

## Data Availability

The datasets presented in this study can be found in online repositories. The names of the repository/repositories and accession number(s) can be found in the article/supplementary material.
